# Bargaining power, decision making, and biofortification: The role of gender in adoption of orange sweet potato in Uganda^☆^

**DOI:** 10.1016/j.foodpol.2020.101909

**Published:** 2020-08

**Authors:** Daniel O. Gilligan, Neha Kumar, Scott McNiven, J.V. Meenakshi, Agnes Quisumbing

**Affiliations:** a International Food Policy Research Institute, United States; b University of California, Davis (posthumous), United States; c Delhi School of Economics, India

**Keywords:** Gender, Bargaining power, Sole and joint decision making, Technology adoption, Biofortification

## Abstract

We examine the role of gender dimensions of intrahousehold bargaining power and decision making in the adoption and diffusion of orange sweet potato (OSP), a biofortified crop being promoted to increase dietary intakes of vitamin A in Uganda. We use patterns of ownership and control of land and other assets by married men and women to create gender-disaggregated indicators of bargaining power, allowing for joint and sole ownership and control of land and assets. Using data from an experimental evaluation of a project promoting OSP adoption, we find that the probability of adopting OSP is not affected by the exclusive or joint control of assets by women at the household level. However, within households, parcels of land under joint control, in which the woman has primary control over decision making, are significantly more likely to contain OSP. Women who control a higher share of household nonland resources are more likely to share OPS vines, showing that women use greater bargaining power to facilitate diffusion of this health-promoting technology. We do not find any impact of women’s bargaining power on children’s dietary intakes of Vitamin A, possibly because husbands and wives have the same preferences regarding their children’s nutritional status. These results contribute to reshaping our understanding of household decision making to inform the design and implementation of agriculture-nutrition interventions.

## Highlights

• Examines gender aspects of intrahousehold bargaining power (BP) in adoption of orange sweet potato.
• Indicators of BP allow for joint and sole ownership/control of land/assets by married men and women.
• Probability of household OSP adoption unaffected by exclusive/joint control of assets by women.
• Within households, OSP more likely on jointly owned parcels where woman is primary decisionmaker.
• Women who control a higher share of household nonland resources more likely to share OPS vines.
• No impact of women’s BP on children’s dietary intakes of Vit A.
• Results help us understand household decision making to inform agriculture-nutrition programs.

## 1 Introduction

Biofortification is emerging as a potentially significant strategy to reduce micronutrient malnutrition. It involves breeding staple food crops to be a rich source of one or more key micronutrients, such as iron, zinc, and vitamin A, and disseminating these crops in areas where micronutrient deficiency is high and where poor households consume a large share of calories from staple foods (Bouis, 2002, Bouis et al., 2011). The success of biofortification as a public health intervention relies on a large share of households substituting conventional varieties of the low-nutrient staple food crop for the biofortified nutrient-dense variety. In many areas in rural Africa and South Asia, where poor households operate near subsistence and have poor market access, getting biofortified food into the diet means fostering broad adoption of the new crop varieties (Gilligan, 2012).

Until fairly recently, agricultural extension efforts assumed that households acted “as one,” consistent with a unitary model of the household in which individuals pooled resources and shared the same preferences (Becker, 1965, Becker, 1981). With empirical evidence often rejecting the unitary model in favor of the collective model of the household (Alderman et al., 1995, Haddad et al., 1997, Quisumbing and Maluccio, 2003), greater attention has been given to the role of intrahousehold decision making and gender dimensions of control of resources in the design of extension programs that aim to increase adoption of new technologies. Recognizing women’s roles both as farmers and decision makers regarding child nutrition, extension messages regarding nutrition are now increasingly targeted to women. Yet, not much is known about the intrahousehold dynamics underlying the adoption and diffusion of new crop varieties, which may affect the success of efforts to disseminate biofortified crops.

We study the role of gender and bargaining power in the adoption and diffusion of biofortified orange sweet potato (OSP), in a project that disseminated OSP to 10,000 households in Uganda from 2007 to 2009. In 2007, the HarvestPlus “Reaching End Users” (REU) project introduced OSP with the goal of increasing dietary intakes of vitamin A and reducing the prevalence of vitamin A deficiency. OSP is a dense source of vitamin A. It is moderately higher yielding than conventional white or yellow sweet potato varieties but is more vulnerable to rot during dry periods. The REU project provided a free one-time distribution of 20 kg of OSP vines to members of selected farmer groups; trainings on OSP cultivation and marketing; limited support on marketing of OSP roots; and trainings for women on the nutritional benefits of consuming OSP and other vitamin A sources.

This paper has three objectives. The principal objective is to analyze how household heads’ and spouses’ bargaining power and decision making may have influenced OSP adoption. A secondary objective is to examine whether women and men in spousal pairs differentially contributed to OSP diffusion, through farmer-to-farmer exchange of planting material. Finally, we examine whether bargaining power mediates the impact of the REU intervention on dietary intakes of Vitamin A among beneficiaries.

We use a broad definition of bargaining power that encompasses the stock of human, natural, and physical assets that men and women control (Quisumbing and Maluccio, 2003; Johnson et al., 2016). Some of these assets are predetermined at the start of the intervention, while others can be affected by the intervention and may influence subsequent intrahousehold bargaining.

This paper expands the analysis of an experimental impact evaluation of the REU project to focus more closely on how gender roles and intrahousehold bargaining may mediate the outcomes of the intervention. The evaluation showed that the REU project led to adoption of OSP by 65% of project households, compared to just five percent in the control group (de Brauw et al., 2018). The project also led to improvements in diet and nutritional status: the intervention reduced the prevalence of inadequate dietary intakes of vitamin A among children under 3 years by 32 percentage points (from a base of 48% dietary inadequacy) and reduced the prevalence of low serum retinol (retinol < 1.05 μmol/L) among children age 3–5 years with low serum retinol at baseline by 9.5 percentage points (Hotz et al., 2012).

In order to better understand the success of this initial introduction of OSP, it is necessary to examine the gender dynamics of intrahousehold decision making in order to find the most cost-effective strategies to promote OSP, so that households will continue to grow it and feed it to their children. The gender dynamics that matter most in these households are those involving spousal pairs—between the wife of the male head of household and her husband, and between the husband of the female head of household and the female head—even if other women and men within these households may also have decision-making influence. Because we have data on bargaining variables only for the wife/female head and the husband/male head, we use these as measures of bargaining power of women and men, restricting the sample appropriately in our analysis.^1^ In this paper, we document the extent to which women play a role in making decisions about which crops the household will grow. We also explore whether OSP adoption, and dietary intakes of vitamin A, are lower in households where women have less bargaining power. If women play a leading role in deciding whether to grow this nutritious crop and those with lower bargaining power have less influence in these decisions, this suggests that men should also be targeted with OSP vines and promotional campaigns about the benefits of OSP consumption. Targeting may be particularly important in households in which women have lower bargaining power due to less education or lower income.

In Uganda, our survey data document that women play an active role in crop selection (particularly for food crops) and commonly supply labor on household farms. Complementary qualitative research (Behrman, 2011) confirms that women take the lead in deciding what food is prepared for the household, particularly for children. Because of this familiar pattern of gender-based specialization in managing child diets, the REU project implementation team decided only to target women for the nutrition trainings, arguing that this would be most cost-effective. Although the biofortified OSP varieties were expected to achieve higher yields than conventional white and yellow sweet potato varieties, the project’s promotional messages emphasized the relative health benefits of OSP, particularly for children and women.^2^ This suggests that, although men and women likely coordinated efforts on the decision to adopt OSP, women may have played an essential role. We attempt to assess the extent to which the bargaining power of women enabled OSP adoption at two levels. First, we model the decision to adopt at the household level. Second, we examine whether our conclusions vary if intrahousehold gender differences are considered. In parcel level analysis, we assess if OSP adoption varied depending on the extent of women’s control over the parcel. We also take into account relevant aspects of women’s human and social capital (knowledge of vitamin A, whether she is a farmer group leader) in modeling the adoption decision.

The speed of diffusion is central to the success of any agricultural intervention where OSP vines are distributed. For many seed crops, adoption can be encouraged through marketing campaigns, but for vegetatively-propagated crops such as sweet potato, planting material such as vine cuttings cannot be stored, making marketing ineffective as a primary dissemination strategy. Instead, most households obtain planting material for these crops through interaction with other households. Indeed, the market for OSP vines was limited at the start of the project. The project offered limited trainings on marketing to encourage households to sell their OSP vines commercially, but markets for OSP only began to develop in one of the three project districts. In contrast, we document that sharing of planting material was common – on average each beneficiary household gave OSP vines to at least one other household. Understanding what factors, including bargaining power, were conducive to such diffusion has implications for the design of cost-effective adoption strategies.

As noted earlier, the REU resulted in improving dietary intakes of Vitamin A among young children. We want to analyze further whether bargaining power mediates not just adoption and diffusion, but also improved nutrition. Vitamin A deficiency causes night blindness and contributes to child morbidity and mortality. Globally, vitamin A deficiency afflicts 127 million young children (West, 2002) and is responsible for six percent of deaths of children under five (Black et al., 2008), and affects 28% of children under age 5 in Uganda (UBOS and ORC Macro, 2001). Understanding how bargaining power and cooperation within the household affect the decisions to adopt a crop that could potentially reduce vitamin A deficiency is of interest to policymakers, designers, and implementors of nutrition-sensitive agricultural projects.

This paper is organized as follows. Section 2 presents a conceptual framework showing the impact pathways underlying the REU project, augmented to consider the potential influence of bargaining power on decisions to grow OSP, to share OSP vines, and on children’s vitamin A intake. Section 3 describes the REU OSP project, the impact evaluation and survey design, and the empirical specification. Section 4 presents the results, and Section 5 includes the discussion and policy implications, respectively.

## 2 Bargaining power and biofortification: Impact pathways

**Fig. 1:**
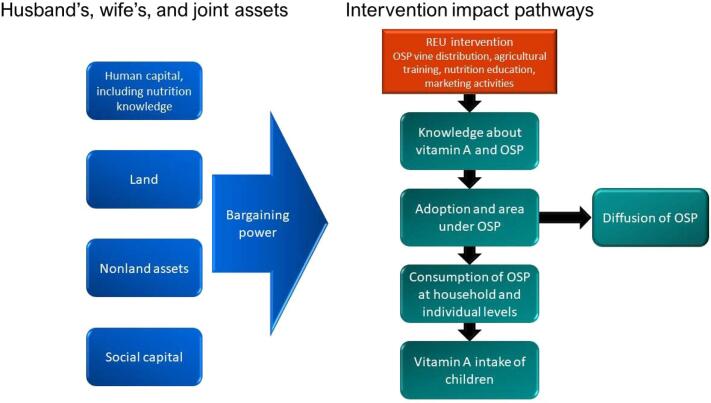
Conceptual framework linking gender, bargaining power, and biofortification.

*Source:* Adapted from de Brauw et al. (2010).

De Brauw et al. (2010) develop a conceptual framework tracing the pathways through which the introduction of OSP by the REU project can affect the prevalence of vitamin A deficiency (right side of Fig. 1). Farmers first learn about OSP, initially through interaction with promoters linked to the agricultural extension program, which includes both agronomic advice and nutrition promotion activities, delivered through farmer groups. Farmers then decide whether to grow OSP, and how much area to devote to OSP rather than traditional sweet potato varieties. Other members of the community may also gain access to OSP, either by purchasing vines, receiving them when shared by other households, or consuming OSP obtained from purchase or gifts. Once OSP is harvested, households decide how much to consume and who will consume it, as influenced by the nutrition promotion activities that aim to increase demand for OSP and other sources of vitamin A. This is intended to increase vitamin A consumption and improve nutritional status of children and mothers.

This conceptual framework (de Brauw et al., 2010) does not allude to the possible role that gender and bargaining power between spouses may play in the decision to grow, consume, and spread OSP. A substantial body of empirical evidence rejects the unitary model of the household, which assumes that household members share the same preferences and pool household resources (Haddad et al., 1997, Schultz, 2001). An alternative, the collective model, allows for the possibility of disagreement between household members, where the resolution may depend on the relative bargaining power of individuals within the household (Manser and Brown, 1980, McElroy and Horney, 1981). Although tests of the bargaining model of the household have typically focused on economic resources that individuals control (Quisumbing and Maluccio, 2003), in this paper, we use a broader definition of bargaining power that encompasses the stock of human, natural, and physical assets that men and women control (Johnson et al., 2016), and account for the possibility that assets may be individually or jointly controlled by spouses (Doss et al., 2014). The expansion of the framework beyond the individually-controlled assets in most bargaining models reflects recent empirical work that suggests that household models that only focus on sole ownership or control of assets may neglect that the substantial portion of household land and assets that is jointly controlled in many societies, including Uganda (see, for example, Doss et al., 2015 on land in Africa). Some of these assets (land, nonland assets, pre-existing nutrition knowledge and farmer group membership) are predetermined at the start of the intervention, while others can be affected by the intervention (nutrition knowledge) and may influence subsequent intrahousehold bargaining.

We expand this conceptual framework to focus on the possible influence of gender roles and bargaining power at different points along the impact pathways (left side of Fig. 1). The four steps outlined in the original framework could then be modified by gender roles and intrahousehold bargaining as follows. The decision to grow OSP could be affected by who owns the land, and who participates in decisions regarding what crop to grow. Decisions regarding what to grow could be affected by bargaining power, proxied by land ownership or ownership of nonland assets, as well as knowledge of the benefits from consuming vitamin-A rich foods. Once OSP is grown, farmers could decide whether to share OSP vines with others; vine-sharing could be affected by the bargaining variables mentioned above, as well as social capital, particularly leadership positions in a farmer group. Diffusion of OSP can be made more effective by not only encouraging the sharing of vines but also providing information about them. Decisions to consume OSP and to give it to specific household members (children) could similarly be affected by intrahousehold bargaining and would be reflected in vitamin A intakes of children.

## 3 Methodology

### 3.1 The Reaching End Users (REU) Orange Sweet Potato (OSP) project

The HarvestPlus REU OSP project disseminated OSP to roughly 10,000 farm households in Uganda, covering four agricultural seasons between August 2007 to August 2009. In Uganda, the first season (February-July) is characterized by heavier rains than the second season (August-December). Through pre-intervention (baseline) and post-intervention (endline) surveys, the project assessed OSP adoption rates and whether adoption resulted in improved vitamin A intakes among young children and their mothers.

The OSP intervention included four components:^3^

(i) a one-time free OSP vine distribution to project households in August 2007,
(ii) agricultural extension services related to OSP production practices and marketing opportunities given to men and women who were members of project farmer groups,
(iii) nutrition training, particularly about vitamin A, given to women in these same households, and
(iv) modest marketing activities for OSP roots and processed products.

Several other aspects of the project and the sample could shape the gender dimensions of OSP adoption. For example, at baseline, nearly sixty percent of farmer group members in the project were women (Table 1). Also, all households in the evaluation sample included at least one household member age 3–5 years old to serve as the primary reference group for dietary assessment.^4^ Because all households in the sample have young children, the age distribution of adult household members is younger than the overall population. However, fertility rates in Uganda are high and many young couples reside with the husband’s parents, so the age distribution of women involved in crop choice decisions in the sample is wide. The average age of women in the sample is 33.5 years (Table 1).

**Table 1:** Gender composition, adoption of OSP, and asset ownership at baseline, analysis sample, 2007.

	Analysis sample
*Gender composition of the sample*	
Share of farmer group members	0.600
who are women, 2007	(0.236)
Age of the woman, 2007 (years)	33.5
	(8.99)

*OSP adoption patterns over time*	
Share of project households adopting OSP	
Season 1	0.916
Season 2	0.879
Season 3	0.800
Season 4	0.663

*Intrahousehold bargaining variables*	
Share of value of land owned in 2007 controlled:	
…exclusively by the woman	0.161
	(0.328)
…exclusively by the man	0.573
	(0.466)
…joint control	0.266
	(0.441)
Share of value of nonland assets in 2007 controlled:	
…exclusively by the woman	0.206
	(0.311)
…exclusively by the man	0.488
	(0.406)
…joint control	0.321
	(0.416)
No. of households	775

Notes: Estimates are means (standard deviations) over farmer group member households in treated farmer groups. Woman refers to the female head/spouse of male head. Man refers to the male head/spouse of female head.

### 3.2 Evaluation design and survey data

The impact evaluation sample includes 84 farmer groups from three districts: Kamuli, Bukedea, and Mukono where white and yellow sweet potato is commonly grown and consumed. Farmer groups were sampled from a list of active farmer groups stratified by district. These farmer groups and households in the village that is home to the largest number of its members represent the sample clusters in the data. Forty-eight of these farmer groups were randomly assigned to the treatment group, and 36 to the control group.

Households with children 3–5 years of age (36–71 months) were sampled, with 14 households per cluster based on power calculations.^5^ The sample also included younger children, aged 6–35 months, to assess the impact of the interventions on their dietary intake of vitamin A. In most farmer groups, the younger siblings of the primary reference children were sampled. In some farmer groups, an additional household was added to the sample to reach the target number of children age 6–35 months. The estimation sample used in this paper consists of 775 households for which we have complete data. Among the 775 households, 415 were treated and 360 were control households.^6^

Data collection took place in two survey rounds, a baseline survey in 2007 and an endline survey in 2009. Both rounds included a detailed socioeconomic survey and a nutrition survey, including a detailed 24-hour dietary recall module. The attrition rate was 7.6% over the two-year period, which is reasonably low relative to other panel surveys (Alderman et al., 2001).

The profile of OSP adoption over the four seasons of the project among participant households is shown in Table 1. Adoption rates of the crop were very high (91.6%) in the first season of the project when 20 kg of free planting material was distributed to all project households.^7^ However, average adoption rates declined in each of the next 3 seasons. Farmers disadopted OSP because their vines dried up and they were unable to obtain new planting material, they did not have sufficient labor to continue to grow the crop (which may reflect the demands of participating in the project or the labor needed to implement the new cropping techniques), or they decided they did not like the crop. There were district-specific patterns: the crop remained very popular in Kamuli and Mukono districts, where 80–85% of project households continued to grow OSP in the fourth season of the project. Most disadoption took place in Bukedea district, where the OSP adoption rate fell to 41.4% in season 4. We therefore include district dummies in all our regression models of OSP adoption.

### 3.3 Measures of bargaining power

We use two main measures of bargaining power to examine how intrahousehold gender relations affect OSP adoption decisions. Although Quisumbing and Maluccio (2003) use aggregated land and nonland assets as a proxy for bargaining power in models testing the unitary household model and the Pareto-efficiency of household decision making, these two forms of wealth may have different effects on intrahousehold decision making.^8^ In particular, we expect that land and nonland assets may play different roles in the decision to adopt the nutritious OSP crop. Women’s access to land may represent both greater bargaining power and an ability to directly affect crop choice and the decision for the household to adopt this nutritious crop. Our first measure of bargaining power is therefore the share of land controlled by the wife of the household head or the female head at baseline (approximately 90% of the household heads are male). This measure directly relates to the relative control of the primary female decision makers in making crop choice decisions. However, access to land in Uganda is usually determined by clan. When a woman marries and joins her husband’s family, her land rights are often restricted if she is not from the same clan (Behrman and Bomuhangi, 2011). Thus, women’s control over land likely represents a form of bargaining power that changes more slowly and is more restricted than one based on control over nonland assets. The second measure is the share of the value of nonland assets at baseline that the woman owns. Women who own a larger share of household assets may have greater discretion over household decisions or stronger bargaining power to win concessions from their male partners. Although there are other women and men in the household, we only have information on the land and nonland assets of spouse; we therefore analyze women’s bargaining power relative to that of husbands/male heads. Using these measures of bargaining power, we estimate the role of gendered ownership and control of land and nonland assets in a household-level model of the determinants of OSP adoption.

We also examine the role of gender in parcel-level models of adoption, based on information on the sex of the decision-maker over each parcel of land. In particular, we differentiate the effects of gender on crop choice by whether crop decisions on a parcel are under the sole control of a man, whether they are solely controlled by a woman, or whether the parcel is under joint control, with either the man or the woman taking the lead in making decisions regarding that parcel. In addition, we also consider the role of human capital, embodied in nutrition knowledge, and social capital, proxied by leadership of a farmer group and the number of years that the household belonged to a farmer group.

### 3.4 Empirical specification

To test the hypothesis that OSP adoption is affected by spouses’ bargaining power, we estimate an adoption equation using a logit specification:

(1) $$P\left({\mathrm{OSP}}_{i}|{\mathrm{covariates}}_{i}\right)=\Lambda \left(\alpha_{0}+\alpha_{1}T_{i}+\alpha_{2}B_{i}+\alpha_{3}T_{i}\times B_{i}+\alpha_{4}C_{i}+\alpha_{5}L_{i}+\alpha_{5}A_{i}+\varphi_{s}Z_{i}\right)$$

where $\Lambda \left(\cdot \right)=exp\left(\cdot \right)/\left[1+\exp\left(\cdot \right)\right]$ is a logistic function.

*P(OSP_i_)* is the probability that household i planted OSP,^9^ conditional on the covariates listed below. T is a binary variable indicating whether the household was assigned to the treatment group; ***B*** is a vector of proxy measures of bargaining power; C is a vector of household characteristics; L is a vector containing information on land and its quality; A is a vector of other attributes that could affect adoption; and Z is a vector of season and district dummies (subscripts omitted).

The measures of bargaining power were constructed using gender-differentiated data from the survey on asset ownership and control over land. This is consistent with other studies using land and assets as measures of bargaining power (e.g. Doss, 1999, Quisumbing and Maluccio, 2003, Fafchamps et al., 2009). For each asset in the baseline asset module, respondents were asked what proportion of the value of the asset was jointly owned, owned only by the household head, or owned only by the spouse of the household head.^10^ Similarly, respondents were asked which household member made the crop choice decisions on each plot, allowing for up to two responses. The data on asset ownership were used to create estimates of the share of land and nonland assets exclusively owned by the woman, exclusively owned by the man, or jointly owned. We use baseline values of these variables so that our measures of bargaining power would be exogenous, or at least predetermined, in the decision to adopt OSP. To test whether bargaining power mediates the impact of the treatment, we interact the bargaining power proxies with the treatment dummy.

In addition to these household level models, we also model OSP adoption at the parcel level given recent findings that productivity gaps exist between male- and female-managed plots in many countries in sub-Saharan Africa (Kilic et al., 2015) and acknowledging that adoption decisions may differ depending on whether the parcel is controlled exclusively by a woman or a man or is jointly controlled. Moreover, household-level analyses tend to underestimate the extent of gender differences in agricultural productivity (Peterman et al., 2011). Specifications at the parcel level also follow equation (1), except that data are allowed to vary at the household level, *i*, and at the parcel level, *j*, for variables on bargaining power, ***B_ij_*** and on land characteristics, ***L_ij_***.

The dependent variable in Section 4.1 is whether the household adopted OSP (at the household and parcel levels) and the share of land planted to OSP, while those in 4.2, 4.3 are whether the household shared planting material and the change in dietary intakes of Vitamin A, respectively.

The measures of relative bargaining power within the household are summarized in Table 1. The woman has exclusive control of only 16% of land assets and 21% of other assets. Respondents reported that 27% of land assets and 32% of nonland assets were jointly owned by both men and women.

We control for other household characteristics in 2007, including: whether the household was headed by a woman, household size, quintiles of per capita expenditure (with the bottom quintile excluded), total land area, whether the household had access to a lowland parcel, the share of “good” or fertile soils. We also include indicators that may indicate a potentially higher probability of adopting OSP, as of 2007, including previous experience in growing OSP, whether the respondent ever changed farming practices as a result of advice received, whether the mother knew what vitamin A was at baseline, whether the mother has access to any radio, whether the respondent is a farmer group leader, the number of years as a farmer group member, the share of sweet potato in planted area, and whether the respondent ever gave farming advice. These values are all evaluated at baseline so that they would be exogenous to extension messages given during the project. Note that because nutrition training was only provided to women, coefficients on nutrition knowledge reflect both the effect of the increased nutrition knowledge as well as differences in preferences. District dummy variables are included, with Kamuli as the excluded category. Appendix Table A1 provides summary statistics. We estimate these equations using a logit specification as detailed above in 4.1, 4.2 and report marginal effects. In one of the parcel-level specifications, we also estimate a conditional logit model, in order to account for the possible correlation of decisions across parcels.

## 4 Results

### 4.1 Bargaining power and adoption of OSP

To provide an overview of the factors affecting the determinants of OSP adoption, we take advantage of the experimental design by first estimating the household-level probability of adopting OSP over our estimation sample of treatment and control households, estimated both for all four seasons of the project (from second season 2007 to first season 2009) and for the fourth season only (first season, 2009). The logit models, shown in Table 2, are estimated using two different sets of household-level measures of bargaining power. Columns (1) and (2) include the land-based measures: the fraction of the land area exclusively owned by the woman, the fraction that is jointly owned and each of these interacted with the treatment variable. Column (1) estimates use data for all four seasons of the project (from second season 2007 to first season 2009) while Column (2) pertains to the fourth season only. We present separate estimates for the fourth season of the project to determine whether the effect of bargaining power is greater after the initial period of experimentation with the new crop. Alternatively, columns (3) and (4) use the value of nonland assets as measures of bargaining power: the fraction of nonland asset value that is exclusively owned; jointly owned; and each interacted with the treatment variable. We treat the land and nonland measures separately, as they are highly correlated, so the regression is uninformative when they are included together. Other controls include household characteristics, background factors affecting adoption, and district dummies.

**Table 2:** The effect of bargaining power between men and women on OSP adoption.

Dep Var: Grow OSP in this season	Woman’s bargaining power based on control over land, seasons 1–4	Woman’s bargaining power based on control over land, season 4	Woman’s bargaining power based on control over nonland assets, seasons 1–4	Woman’s bargaining power based on control over nonland assets, season 4
	(1)	(2)	(3)	(4)
Treated	0.461***	0.508***	0.463***	0.536***
	(0.025)	(0.055)	(0.030)	(0.055)
Fraction of land exclusively	−0.029	−0.083		
owned by the woman in 2007	(0.082)	(0.147)		
Fraction of land exclusively	0.042	0.100		
owned by the woman **×** Treated	(0.078)	(0.142)		
Fraction of land jointly	0.007	0.077		
owned in 2007	(0.037)	(0.068)		
Fraction of land jointly owned × Treated	−0.002	−0.074		
	(0.041)	(0.074)		
Fraction of value of nonland assets exclusively			−0.030	−0.119
wned by the woman in 2007			(0.075)	(0.125)
Fraction of value of nonland assets exclusively			0.037	0.046
owned by the woman **×** Treated			(0.073)	(0.121)
Fraction of value of nonland			−0.008	0.074
assets jointly owned			(0.039)	(0.068)
Fraction of nonland assets jointly			−0.013	−0.125*
owned × Treated			(0.043)	(0.073)
Observations	3100	775	3100	775

Notes: Models are logit models. Columns (1) and (3) present models estimated over 4 seasons from 2007 to 09. Columns (2) and (4) present models estimated for the fourth season of the project only. All models include the following baseline control variables: female headed household, household size, household head education, quintiles 2–5 of total household expenditure per adult equivalent, total land area owned, whether the household had access to lowland parcels, share of land with ‘good’ soil quality, any prior experience growing OSP, ever changed farming practices in response to advice, whether mother knows what vitamin A is, whether mother has access to any radio, if household includes a farmer group leader, number of year as a farmer group member, share of sweet potato in planted area, whether household ever gives farming advice, and season and district indicator variables. Sample is farmer group member households in treated and control farmer groups and includes all households from the panel with complete data on all variables used in this paper *significant at the 10% level; **significant at the 5% level; ***significant at the 1% level.

In all specifications, the coefficient on the treatment dummy is significant and positive, a reflection of the successful dissemination effort of the REU project. However, none of the bargaining power variables—the fraction of land and nonland assets exclusively or jointly owned—are significant.^11^ Interacting these with the treatment variables also does not help distinguish any treatment heterogeneity. These results would seem to support the unitary model: the two measures of women’s control over wealth do not seem to translate into differential effects on the decision to adopt OSP.^12^

However, analysis at this level of aggregation may mask a more complex decision-making process occurring within households. Most households have access to at least two parcels of land for farming and may have worked out an implicit agreement over which household members control crop choice and farming decisions on each parcel. For a particular parcel, the crop choice decision may be a joint decision by the household head and spouse, or a particular household member may maintain sole control over the parcel. However, a household member with sole control over a parcel may still consider the crops being grown on other parcels when making crop choice decisions for that parcel. Although several recent papers have highlighted the importance of intra-household analysis of agricultural productivity, the evidence on whether there are gender differentials in crop productivity, and the sources of these differentials is mixed and context specific. Variables such as input use intensity, security of land tenure, and access to resources are all important determinants of such differentials; recent estimates suggest that yield differences between male- and female-managed plots not only come from differences in control of resources, but also from differences in returns to a given bundle of resources (see for example, Karamba and Winters, 2015, Kilic et al., 2015, Oseni et al., 2015).

Our data allow us to differentiate the gender dimensions of the control over decision making at the parcel level. We therefore undertake a parcel-level analysis of adoption decisions. The survey asked, “Who decided what to grow on this parcel?” in the first season of 2009. Respondents were allowed to give up to two responses. We interpret the order of household members listed as indicating which household member played a larger role in the crop choice decision. The most common arrangement, on nearly 61% of parcels, is one in which control over crop choice is joint, but that a man takes the lead in making this decision. These parcels are likely to be larger, with better soil quality, and are the household’s primary asset for agricultural production; it is not surprising that decisions on this parcel are made jointly. Women are sole decisionmakers on 17% of parcels; although this accounts for all (but one) of the female headed households in this sample (which are primarily single-headed households headed by women), solely women-managed plots are also found in about 19% of the male headed households. This may partly be due to women having sole control of plots that are of lower quality or with smaller areas. Only 4.5% of parcels are reported to be controlled exclusively by a man, while the remaining 16.5% of parcels are under joint control with a woman taking the lead in the decision making.

At the parcel level, the probability of adoption of OSP in 2009 is higher for parcels controlled exclusively by a woman than for parcels exclusively controlled by a man or under joint control but with a man taking the lead, as shown in Table 3. Similarly, OSP adoption is significantly more likely on parcels under joint control in which a woman is the primary decisionmaker than on jointly-controlled parcels with a man leading the crop choice decision. This pattern of behavior is quite different when considering land area devoted to OSP. The share of area planted under OSP is highest on parcels under joint control, but with a man leading decision making (at 13.8% of cultivated area). However, this share of area under OSP is not significantly different for parcels with joint control in which a woman leads decision making. In fact, area under OSP is lowest on parcels exclusively controlled by a man. These patterns are informative, but they do not control for a variety of factors that account for selection into parcel control within the household or the joint decision of the household concerning what to grow on all of its parcels.

**Table 3:** Mean probability of OSP adoption and area planted by gender of decision maker and type of decision making.

Dep var: “Who decided what to grow on this parcel?”	Women only	Men only	Joint, women first	Joint, men first
	(1)	(2)	(3)	(4)
Grow OSP on this parcel	45.1^a^, ^c^	26.1^b^	48.3^c^	35.3
Share of parcel area planted with OSP (unconditional)	0.128^a^, ^b^	0.076^c^	0.095^c^	0.138
Parcel area planted with OSP (acres)	0.079^a^, ^c^	0.045^b^, ^c^	0.093^c^	0.104

Notes: Estimates are averages over all four seasons for farmer group member households in treated farmer groups (n = 415 households).

a Significantly different from (2) “Men only”.

b Significantly different from (3) “Joint, women 1st”.

c Significantly different from (4) “Joint, men 1st”.

We account for these in Table 4, which presents a model of the determinants of the decision to grow OSP at the parcel level by season, controlling for baseline responses on control over parcel decision making by gender. Accounting for these observables matters: the coefficients in column 1 suggest that OSP is significantly more likely to be planted on parcels with joint control, but where a woman was listed first in order of control than on parcels under joint control but where a man is listed first (the omitted category). In a model conditional on whether the household is growing OSP on any parcel (column 2), parcels controlled only by a woman are not significantly more likely to have OSP than those with joint control in which men have primary control, but parcels controlled only by men are significantly less likely to have OSP.^13^

**Table 4:** Effect of gender in control over parcel decisions on OSP adoption over four seasons.

Dep Var: Grow OSP on this parcel	Treated households (Logit model)	Treated households that adopted OSP (Logit model)	Treated households (Conditional logit model)
	(1)	(2)	(3)
Parcel control: women only	0.027	0.018	0.644
	(0.027)	(0.028)	(0.265)
Parcel control: men only	−0.074	−0.147**	0.737
	(0.054)	(0.068)	(0.358)
Parcel control: women listed first	0.058**	0.040	1.171
	(0.026)	(0.025)	(0.213)
Household size	0.002	−0.001	
	(0.004)	(0.004)	
Female headed household	0.017	−0.018	
	(0.038)	(0.038)	
Log of monthly expenditure per adult equivalent	0.028**	0.012	
	(0.013)	(0.017)	
Mother’s knowledge of vitamin A, 2007	0.059***	0.041**	
	(0.014)	(0.016)	
Change in mother’s knowledge of	0.049***	0.036***	
vitamin A, 2007–2009	(0.012)	(0.013)	
Share of sweet potato in land area, 2007	0.213***	0.110*	
	(0.061)	(0.058)	
Total land area operated in this season, acres	−0.057***	−0.061***	0.673***
	(0.007)	(0.009)	(0.052)
Household member is farmer group leader	0.065***	0.037	
	(0.022)	(0.027)	
Distance to FG meeting place	−0.000	0.000	
	(0.001)	(0.001)	
Ln of farmer group size, 2007	−0.102	0.002	
	(0.066)	(0.061)	
Parcel area, acres	0.126***	0.144***	1.496***
	(0.013)	(0.016)	(0.127)
Parcel has good soil, 2009	0.002	0.006	1.319*
	(0.015)	(0.021)	(0.209)
Parcel tenure status, freehold, 2009	−0.234***	−0.646*	0.680
	(0.072)	(0.345)	(0.477)
Season 2	0.022*	0.067***	1.136
	(0.013)	(0.017)	(0.087)
Season 3	−0.012	0.041**	0.874*
	(0.014)	(0.018)	(0.069)
Season 4	−0.114***	0.026	0.524***
	(0.018)	(0.019)	(0.057)

Observations	3707	2283	3308
Households in estimation sample	415	275	339

Notes: Dependent variable is 1 if OSP grown on this parcel in this season, 0 otherwise. Estimates in columns (1) and (2) are marginal effects at the mean of the data from logit models. Column (3) presents odds ratios and linearized standard errors in parentheses from a conditional logit model. Household-level variables drop from this model as do parcel observations from households in which OSP is grown on all parcels or none of the parcels. Sample is farmer group member households in treated farmer groups. Omitted category for Parcel Control is joint, male head/husband listed first. The sample in column 1 includes observations at the parcel level (on average, just over 2 parcels per household) for four seasons. Results in column 2 are restricted to treated households that adopted OSP on at least one parcel, and are also at the parcel level for all four seasons. The conditional logit model in column 3 drops households that have only one parcel-level observation. Standard errors adjusted for stratification by district and clustering at the farmer group level.

* Significant at the 10% level.

** Significant at the 5% level.

*** Significant at the 1% level.

It is important to keep in mind that these effects of bargaining power on parcel-level decisions to grow OSP do not capture the effect of bargaining power on prior negotiated assignment of decision makers to parcels. Rather, these estimates show that, conditional on those assignments, OSP is more likely to be grown on jointly managed parcels where women exert greater control. This provides evidence that, within households, either women are more likely to choose to grow OSP when they have the opportunity to do so (on plots they control) or that men and women agree to grow OSP and the crop ends up on women-controlled plots as a result of intrahousehold specialization of tasks. Either interpretation documents substantial cooperation between spouses as well as the importance of women’s (shared) control over farming decisions.

These models also provide evidence of other factors shaping the OSP adoption decision. In both columns 1 and 2, the probability of adopting OSP on a parcel increases significantly with mother’s nutrition knowledge—the coefficients associated with the number of facts related to vitamin A that the mother of the reference child knew in baseline and the change in her knowledge over the course of the REU project are both significant. The probability of adopting OSP also increases significantly with the share of land area that the household had planted with sweet potato (white, yellow or orange) on all of its parcels at baseline. This suggests that households are substituting area under production with white or yellow sweet potato with OSP, as anticipated by the biofortification program. OSP adoption is less likely on farms with larger land holdings, but conditional on total landholdings, OSP is more likely to be grown on parcels with more land area. Land tenure also affects crop choice decisions: parcels under the free hold land tenure status are less likely to be selected for adopting OSP, although this relationship is only weakly significant in column 2. Free hold tenure arrangements provide greater security of land tenure than the more common customary or *mailo* arrangements. *Mailo* is a form of land tenure that provides rights to occupants of land owned by someone else; it is common in the Buganda region of Uganda (Mukono district). Consistent with other evidence from Uganda that more secure tenure creates incentives to plant permanent crops (Deininger et al., 2008), farmers may be selecting crops that require more investment in land or take longer to mature on free hold parcels, given that OSP vines can be easily transplanted to other parcels.

None of the models presented so far account for potential correlations of decisions of what to grow across parcels within the household. When we account for this in estimation (column 3) using a conditional logit model, the pattern of effects remains similar. Results in column 3 present the odds ratio of the probability of adopting OSP compared to parcels under joint control with a man leading decision making. The point estimates suggest that the probability of adopting OSP is highest on parcels with joint control and a woman leading decision making and lower on parcels under control of a single gender, but these estimates are not significant. One reason for the insignificance may be the smaller sample in column 3 (households with only one observation are dropped in the conditional logit model) than in column1. As mentioned earlier, the estimation sample was harmonized to eliminate item nonresponse across all tables. Results from estimating the specifications in Table 4 on a larger sample (see Appendix Table A2) show stronger effects of the bargaining power variables. In particular, in column 3, the coefficient associated with a woman leading decisions on a parcel under joint control is significant at the 5% level.

### 4.2 Bargaining power and OSP diffusion

The cost-effectiveness of the REU project as a biofortification strategy to improve dietary intakes of vitamin A would be greatly improved if households in the project shared OSP planting material with other households. Only a small amount of OSP planting material is needed to start a small plot, so project households could share planting material with others without significantly affecting their productivity. However, the vine cuttings must be transplanted within a day or so, else they wither and die. This feature discourages large commercial operations selling OSP planting material. In our survey, most households at baseline reported receiving their traditional sweet potato vines from neighbors and friends. The potential for this exchange is shaped by the patterns of interactions between households in a community. Women and men have overlapping but often different social or information spheres. An important question is how these gender-differentiated spheres of interaction play a role in OSP diffusion. In related work, McNiven and Gilligan (2012) show that information networks within communities play a substantial role in first providing access to OSP planting material and later in supporting sustained OSP adoption by households outside the project.

On average, each household in the project gave OSP planting material to 1.2 other households during the two years of the project. We examine the role of women and men farmer group members as well as the role of women’s bargaining power in the household’s decision to participate in OSP diffusion. Results, based on the 415 treated households, are presented in Table 5.

**Table 5:** Gender-based differences in diffusion of OSP, 2007–2009

Dep Var: Treated households shared OSP vines with other households	Women’s bargaining power based on control over land	Women’s bargaining power based on control over nonland assets
	(1)	(2)
Household has at least one woman farmer group member	0.011 (0.074)	0.021 (0.076)
Fraction of value of land exclusively owned by the woman in 2007	0.241 (0.149)	

Fraction of value of land jointly owned in 2007	0.060 (0.059)	

Fraction of value of nonland assets exclusively owned by the woman in 2007		0.460*** (0.144)
Fraction of value of nonland assets jointly owned in 2007		0.172**
		(0.069)
Female-headed household	−0.354**	−0.479***
	(0.162)	(0.160)
Household size	0.002	0.003
	(0.010)	(0.011)
Household head education (in years)	0.015	0.021
	(0.074)	(0.075)
Quintile 2: Total expenditure per adult eq.	0.000	0.014
	(0.075)	(0.072)
Quintile 3: Total expenditure per adult eq.	−0.001	0.028
	(0.083)	(0.082)
Quintile 4: Total expenditure per adult eq.	0.110	0.128
	(0.083)	(0.080)
Quintile 5: Total expenditure per adult eq.	−0.012	−0.013
	(0.008)	(0.008)
Total land area, 2007	0.102	0.094
	(0.082)	(0.081)
Woman’s share of land area, 2007	−0.080	−0.082
	(0.060)	(0.059)
Share of ‘good’ soils, 2007	−0.084	−0.081
	(0.057)	(0.056)
Ever changed farming practices as a result	0.397*	0.362*
of advice received	(0.205)	(0.211)
Mother knows what vitamin A is, 2007	0.191**	0.190**
	(0.075)	(0.075)
Farmer group leader	0.150**	0.149**
	(0.068)	(0.069)
Ever give advice on farming, 2007	0.262***	0.292***
	(0.079)	(0.082)
Bukedea	0.268***	0.254***
	(0.078)	(0.074)
Mukono	−0.354**	−0.479***
	(0.162)	(0.160)
Observations	415	415
Average fraction of households that shared OSP vines with other households	0.563	

Notes: Models estimated are logit models. Estimates are marginal effects at the mean of the data. Sample is farmer group member households in treated farmer groups.

* Significant at the 10% level.

** Significant at the 5% level.

*** Significant at the 1% level.

Interestingly, the share of land area controlled by women in the household does not have a significant effect on the probability of conducting diffusion in the full sample. Instead, the share of nonland asset value either exclusively or jointly under the control of women is positively associated with the probability of diffusion. Women who control a higher share of household nonland resources are more likely to share this nutrition-promoting agricultural technology, which is likely to help the women build social capital within their communities. The result that bargaining power based on nonland assets plays a bigger role in prompting women to share OSP vines than the share of land women control may indicate that bargaining power based on land is more restricted by longstanding norms on land rights obtained through marriage, which would have been determined at the time of household formation.

Among households participating in project farmer groups, whether a household has at least one woman farmer group member does not affect the probability of the household undertaking any diffusion during the project. However, households in which the woman was a farmer group leader, or otherwise gave advice on farming in the baseline, were far more likely to share vines with others. Also, irrespective of the specification of women’s bargaining power, the probability of sharing the OSP crop increases significantly if the mother has some knowledge about vitamin A at baseline. These appear to be important channels to promote farmer-to-farmer diffusion and also reflect the woman’s stock of knowledge (human capital) and social capital (due to her leadership in the farmer group).

### 4.3 Bargaining power and dietary intake of vitamin A

Finally, we examine the effect of women’s bargaining power on dietary intake of vitamin A by children in the reference group of 3–5 year olds. Improving dietary intakes of vitamin A for this group of reference children was a major objective of the project. The impacts of the project on dietary intake of vitamin A are reported in Hotz et al. (2012). Here we examine how gender roles shape this result. Dietary intake of the reference children was measured in the survey through a comprehensive dietary recall interview of their mothers. Respondents were asked to list each food consumed by the child in the day before the interview and then were asked about the composition of the foods consumed, for complex dishes. The weight (or volume) of each food consumed by the child was recorded and these were then converted into nutrient values using a detailed food composition table constructed for this study. Table 6 presents results from OLS estimation, regressing the difference in vitamin A intakes between endline and baseline (a continuous variable) on household-level bargaining power variables at baseline. The sample includes the subsample of children age 3–5 years at baseline from the estimation sample households on which dietary recall data were available (n = 327).

**Table 6:** Gender differences in control over assets and child consumption of vitamin A

Dep Var: Change in dietary intake of vitamin A (μg RAE), 2007–2009	Women’s bargaining power based on control over land	Women’s bargaining power based on control over land interacted with treatment	Women’s bargaining power based on control over nonland assets	Women’s bargaining power based on control over nonland assets interacted with treatment
	(1)	(2)	(3)	(4)
Treated	336.293*	404.154	339.691*	439.065
	(174.932)	(259.572)	(175.943)	(346.164)
Fraction of value of land exclusively owned by	330.648	201.128		
women in 2007	(309.972)	(358.380)		
Fraction of value of land exclusively		207.627		
owned by women × Treated		(572.162)		
Fraction of value of land jointly	32.447	250.264		
owned in 2007	(197.177)	(258.663)		
Fraction of land jointly owned × Treated		−376.044		
		(374.360)		
Fraction of value of nonland assets			374.234	382.243
exclusively owned by the women in 2007			(350.158)	(483.458)
Fraction of value of nonland assets exclusively			−31.159	143.126
owned by women × Treated			(236.720)	(348.609)
Fraction of value of nonland				−16.869
assets jointly owned				(674.728)
Fraction of nonland assets jointly				−303.356
owned × Treated				(475.188)
Observations	327	327	327	327

Mean dietary intake of vitamin A (μg RAE), 2007	517.83			

Notes: Models are ordinary least squares models. Units are μg of retinol activity equivalents, a measure of vitamin A in the diet. Sample includes the subsample of children age 3–5 years at baseline on which dietary recall data were available. Other control variables not reported. *significant at the 10% level, ^**^significant at the 5% level, ***significant at the 1% level.

The results show a weakly statistically significant effect of the REU intervention on dietary intake of vitamin A (columns 1 and 3, Table 6) on children age 3–5 years. The effect is large at 65% of mean intakes of children in the control group.^14^^,^^15^ However, none of the bargaining power variables are significant, either singly or when interacted with the treatment. Thus, this may indicate either that women were unable to use their bargaining power to increase the impact of the REU project on child consumption of vitamin A, or that husbands and wives have the same preferences—they both want to improve child nutritional status—and thus individual bargaining variables did not have an impact. Fafchamps et al., (2009), analyzing data from Ethiopia, attribute the lack of significance of bargaining variables to possible externalities: preferences over child outcomes may be too similar across spouses, thereby making it impossible to identify the effect of bargaining variables.

## 5 Discussion and policy implications

We find a complex relationship of women’s bargaining power and control over household assets to the impact of the Uganda OSP biofortification program on OSP adoption, diffusion and dietary intakes of vitamin A. Women’s bargaining power, measured by the share of land and nonland assets exclusively controlled by the wife/female head, does not unambiguously increase the probability that a household adopts OSP in response to the project, possibly because land and nonland assets do not affect OSP adoption in the same way. Land parcels over which the woman has sole control are not those most likely to contain OSP. Rather, the probability of adoption of OSP is highest on parcels for which there is joint control, but where the woman takes the lead in deciding which crops are grown. However, the probability of adopting OSP is also lowest on parcels exclusively controlled by the man among households that planted at least some OSP.

We acknowledge that these estimates do not identify whether the observed effects are due to gender-based differences in preferences, in information or in specialization of activities within households. We find an effect of women’s bargaining power (based on the value of nonland assets owned by the wife/female head) and farmer group participation in the diffusion of the OSP crop technology, but not on Vitamin A intakes. The insignificant impact of bargaining power variables on children’s vitamin A intakes may reflect very similar preferences between spouses for child nutrition, so the treatment effect rather than the bargaining variables dominates. It is also possible that the effects of bargaining power are domain specific.

These results contribute to reshaping our understanding of household decision making, and in turn, the design of agriculture-nutrition interventions. Even if decisions to grow OSP involve only a small portion of the household’s land, these decisions are both economically and nutritionally important. The economic importance of OSP adoption arises from its being a dense source of vitamin A. Low dietary intake of vitamin A is associated with higher child morbidity and vitamin A deficiency is a major contributor to child mortality globally. Efficacy trials have shown that consuming just 125 g of orange sweet potato improved vitamin A status (vitamin A liver stores) in children age 5–10 years in South Africa (van Jaarsveld et al., 2005). Thus, even a small parcel of orange sweet potato can be a major source of vitamin A for the household; understanding the dynamics underlying crop adoption is important for scaling up future interventions.

While substantial evidence supports a non-unitary model of the household, bargaining models have focused on individually consumed goods (or leisure) rather than joint production or consumption of the public good (Doss and Quisumbing, 2020). Neglecting joint decision making overlooks the fact that globally, both sole and joint management and production by men and women take place on family farms. Indeed, our findings indicate that while women play an important role, and often a leading role, in the decision to adopt OSP, this decision is often jointly made with their husbands. Because of the jointness of these decisions, the current strategy of targeting only women with nutritional trainings may be missing an opportunity to create an awareness of the benefits of OSP among men. The evaluation of the REU project found no evidence of impact on fathers’ knowledge of child feeding practices in Uganda (de Brauw et al., 2010), but the contribution of nutrition messages received by women on the impact of the project on OSP adoption and dietary intakes of vitamin A also appears to be relatively small (de Brauw et al., 2018). In this setting, our results suggest that engagement of the project with adult household members of both genders may be the best strategy to promote adoption.

Framing nutrition-sensitive agricultural interventions in the context of cooperation and joint interests, rather than as a zero-sum game, may increase support for such programs, and may eventually lead to the adoption of technologies and behaviors that promote desired nutritional goals and other development objectives. Indeed, recognizing the different preferences of household members and strengthening cooperation within the household is the core of the International Fund for Agricultural Development’s household methodologies approach (International Fund for Agricultural Development, 2019). More recent nutrition-sensitive agricultural projects have taken a more deliberate approach to including men in program activities, such as including them in nutrition training (and including women in agricultural extension training) and undertaking program activities directed towards men and community leaders that attempt to shift gender norms. Emerging results from impact evaluations of nutrition-sensitive agricultural projects in Bangladesh (Ahmed et al., 2018) and Malawi (Gelli et al., 2018) show promise. Ongoing evaluations of this new generation of programs are expected to yield new insights into how gender dynamics affect the adoption of high-value nutrition crops like OSP, and, in turn, update the guidelines for designing and implementing gender- and nutrition-sensitive agricultural projects.

## CRediT authorship contribution statement

**Daniel O. Gilligan:** Conceptualization, Formal analysis, Investigation, Writing - original draft. **Neha Kumar:** Conceptualization, Formal analysis, Investigation, Writing - original draft. **Scott McNiven:** Investigation, Conceptualization . **J.V. Meenakshi:** Conceptualization, Writing - review & editing. **Agnes Quisumbing:** Conceptualization, Investigation, Writing - original draft.

## Figures and Tables

**Table A1:** Summary statistics of harmonized estimation sample (N = 775 households)

Household characteristics	Mean	Standard Deviation
Female-headed household	0.10	0.30
Household size	7.68	2.68
Second quintile of per adult equivalent total expenditure	0.20	0.40
Third quintile of per adult equivalent total expenditure	0.19	0.40
Fourth quintile of per adult equivalent total expenditure	0.21	0.41
Fifth quintile of per adult equivalent total expenditure	0.18	0.39
Total expendture per adult equivalent, (‘000 UGS)	37706.46	30262.17
Total monthly expenditure per capita	27335.34	22371.61
Total land area, 2007	3.28	14.30
Woman’s share of land area, 2007	0.25	0.41
Household has a lowland parcel, 2007	0.43	0.49
Share of ‘good’ soils, 2007	0.51	0.46
Household had ever grown OSP, 2007	0.06	0.24
Ever changed farming practices as a result of advice received	0.60	0.49
Mother knows what vitamin A is, 2007	0.91	0.29
Mother listened to radio last month, 2007	0.58	0.49
Farmer group leader	0.18	0.39
Years of membership with farmer group	2.03	1.89
Sweet potato area share, 1st season 2007	0.15	0.17
Ever give advice on farming, 2007	0.70	0.46

**Table A2:** Alternative results for Table 4.

Dep Var: Grow OSP on this parcel	Treated households (Logit model)	Treated households that adopted OSP (Logit model)	Treated households (Conditional logit model)
	(1)	(2)	(3)
Parcel control: women only	0.011	−0.006	1.050
	(0.027)	(0.030)	(0.301)
Parcel control: men only	−0.133***	−0.203***	0.677
	(0.043)	(0.040)	(0.313)
Parcel control: women listed first	0.070***	0.042*	1.396**
	(0.021)	(0.023)	(0.232)
Household size	0.000	−0.002	
	(0.004)	(0.004)	
Female headed household	0.021	0.002	
	(0.037)	(0.039)	
Log of monthly expenditure per adult equ.	0.021**	0.011	
	(0.009)	(0.015)	
Mother’s knowledge of vitamin A, 2007	0.048***	0.017	
	(0.012)	(0.015)	
Change in mother’s knowledge of vitamin A,	0.041***	0.020	
2007–2009	(0.011)	(0.012)	
Share of sweet potato in land area, 2007	0.183***	0.058	
	(0.053)	(0.047)	
Total land area operated in this season, acres	−0.052***	−0.054***	0.680***
	(0.006)	(0.008)	(0.037)
Household member is farmer group leader	0.057***	0.032	
	(0.021)	(0.025)	
Distance to FG meeting place	0.000	0.001	
	(0.001)	(0.001)	
Ln of farmer group size, 2007	−0.109*	−0.014	
	(0.059)	(0.057)	
Parcel area, acres	0.117***	0.127***	1.396***
	(0.011)	(0.014)	(0.093)
Parcel has good soil, 2009	−0.014	−0.021	1.077
	(0.016)	(0.020)	(0.166)
Parcel tenure status, freehold, 2009	−0.178**	−0.295	0.555
	(0.081)	(0.261)	(0.375)
Season 2	0.019	0.067***	1.110
	(0.013)	(0.015)	(0.081)
Season 3	−0.010	0.046***	0.879*
	(0.013)	(0.016)	(0.067)
Season 4	−0.111***	0.025	0.539***
	(0.016)	(0.015)	(0.049)
	4809	3052	4284
Observations	0.011	−0.006	1.050
Households in estimation sample	555	371	451

Note: The data used in this regression are taken from the full sample, not the harmonized estimation sample, restricted to treatment households where the woman is a farmer group member and the woman and man (the main couple) are the only decisionmakers included in the estimation.

## Appendix

See Table A1, Table A2.

## Footnotes

1 We refer to the female head or the spouse of the male head as the “woman” or “women” and the male head/spouse of female head as the “man” or “men”, for the sake of brevity.

2 An efficacy trial conducted in South Africa (van Jaarsveld et al., 2005) had already demonstrated that consumption of OSP increases dietary intakes of vitamin A and increases serum retinol concentrations, a measure of vitamin A status.

3 The intervention was randomized across two arms, one of which (Model 1) was more intensive that the other (Model 2). While component (i) was identical across the two intervention arms; Model 1 and Model 2 households received 20 kg of OSP vines on average during the same period in 2007. Components (ii) and (iii) were provided for two years in Model 1 and for one year in Model 2, at a savings of roughly 30 percent of total model costs. Because impacts did not differ across these two arms (see Hotz et al., 2012; de Brauw et al., 2018), this distinction is not maintained in this paper; we refer only to the pooled binary treatment and the control group.

4 The sample is unbalanced, with fewer farmer groups in Model 2, because including the large samples required for biochemical assessment to measure serum retinol in all three intervention arms was too costly. Blood samples were only taken in households in the Model 1 and Control groups. Children age 3–5 years at baseline comprised the primary reference group for dietary assessment.

5 This was based on the number needed to detect the minimum effect size desired for serum retinol measured in blood samples, after allowing attrition of two households per farmer group (in the treatment arm and control group where blood samples would be collected). In the other arm, only dietary intake data was elicited, for which the required sample sizes were smaller (12), but we sampled 14 households in these clusters as well to maintain comparability. See de Brauw et al. (2010) for further details on sampling.

6 The panel data from the impact evaluation baseline and endline surveys includes 1,116 households. The estimation sample is restricted to those households for which there was no item nonresponse. Variables with relatively high nonresponse include the bargaining variables on share of assets exclusively or jointly controlled by women, which may reflect some difficulty of responding to these questions. Tests of balance suggest that the estimation sample and overall sample are similar, on average.

7 The 20kg of planting material distributed is sufficient to plant one quarter of an acre of OSP under the planting guidelines taught by the project.

8 In rural societies where imperfect factor markets limit the convertability of assets into different forms, and where land may have an intrinsic social and cultural value, any attempt to aggregate land and other assets using market values may be flawed.

9 We omit the season subscript s for convenience.

10 Information on the value of assets owned by the household was collected during the baseline survey in 2007. However, the questions on what proportion of these baseline assets was under the control of the household head, spouse, or joint control were not asked until the endline survey in 2009. During the endline survey interview in 2009, enumerators reminded respondents of the value of each asset the household reported owning in 2007, and then asked the questions about control of the asset at that time. The respondent for this module was the main farmer- which could be the household head or the spouse. Although there may be some recall bias in remembering who controlled the asset two years before, we believe that in most cases this gender disaggregation in control is fairly stable, which would limit the degree of recall bias. Also, when we use these variables as control variables for OSP adoption decisions we assume that any bias in the recall on gender disaggregated control over these assets is not correlated with the treatment.

11 Only the coefficient in column 4 of Table 2 for the fraction of nonland assets jointly owned interacted with the treatment indicator is weakly statistically significant.

12 There are language differences across the three districts (Luganda is dominant in Mukono district, Teso in Bukedea district and Luganda and Lusoga in Kamuli district), suggesting some potential for cultural differences in bargaining power, but it is not possible to separate out these effects because they largely differ by district. Such district-specific cultural differences are swept up in district dummy variables along with many other district-level effects, but district dummy variables are needed to control for district stratification of randomization in the study design. We can conclude however that our estimates of the effect of bargaining power on OSP adoption are conservative if these cultural effects are important.

13 In these models, however, we cannot disentangle the effect of factors that determine the gender of the controller of the parcel and the gender of the controller on the adoption decision. Such division of parcels between various household members is more of “long-term/one-time” decision as opposed to crop choice which may be more recurrent. It is possible that certain parcels of land are more suited to growing of certain crops that may be deemed as cash crops for the household and are therefore managed by the household head.

14 The effect reported here on the pooled treatment interventions lies between the estimates for adjusted dietary intakes originally reported in Hotz et al. 2012. The effect reported here is only weakly significant, likely due to the smaller sample of households with the complete set of bargaining variables.

15 The average requirement for children age 4–8 years old is 275 μg RAE/day.
